# Influence of microbiological diagnosis on the clinical course of spondylodiscitis

**DOI:** 10.1007/s15010-021-01642-5

**Published:** 2021-07-12

**Authors:** Martin Stangenberg, Klaus Christian Mende, Malte Mohme, Theresa Krätzig, Lennart Viezens, Anna Both, Holger Rohde, Marc Dreimann

**Affiliations:** 1 Division of Spine Surgery, Department of Trauma and Orthopedic Surgery, University Medical Center Hamburg-Eppendorf, Martinistr. 52, 20246 Hamburg, Germany; 2 Department of Neurosurgery, University Medical Center Hamburg-Eppendorf, Hamburg, Germany; 3 ﻿Institute of Medical Microbiology, Virology and Hygiene, University Medical Center Hamburg-Eppendorf, Hamburg, Germany

**Keywords:** Spondylodiscitis, Vertebral osteomyelitis, Microbiology, Risk factors, *Staphylococcus aureus*

## Abstract

**Purpose:**

This study sought to recognize differences in clinical disease manifestations of spondylodiscitis depending on the causative bacterial species.

**Methods:**

We performed an evaluation of all spondylodiscitis cases in our clinic from 2013–2018. 211 patients were included, in whom a causative bacterial pathogen was identified in 80.6% (170/211). We collected the following data; disease complications, comorbidities, laboratory parameters, abscess occurrence, localization of the infection (cervical, thoracic, lumbar, disseminated), length of hospital stay and 30-day mortality rates depending on the causative bacterial species. Differences between bacterial detection in blood culture and intraoperative samples were also recorded.

**Results:**

The detection rate of bacterial pathogens through intraoperative sampling was 66.3% and could be increased by the results of the blood cultures to a total of 80.6% (*n* = 170/211). *S. aureus* was the most frequently detected pathogen in blood culture and intraoperative specimens and and was isolated in a higher percentage cervically than in other locations of the spine. Bacteremic *S. aureus* infections were associated with an increased mortality (31.4% vs. overall mortality of 13.7%, *p* = 0.001), more frequently developing complications, such as shock, pneumonia, and myocardial infarction. Comorbidities, abscesses, length of stay, sex, and laboratory parameters all showed no differences depending on the bacterial species.

**Conclusion:**

Blood culture significantly improved the diagnostic yield, thus underscoring the need for a structured diagnostic approach. MSSA spondylodiscitis was associated with increased mortality and a higher incidence of complications.

## Introduction

Spondylodiscitis is an infection of the intervertebral disc with concomitant vertebral osteomyelitis, and is responsible for 2–5% of all infectious bone diseases [1–3]. The infection mainly develops in the elderly, immune-compromised, and chronically ill patients, with males being affected in approximately two-thirds of cases [4, 5].

Previously, spondylodiscitis frequency was reported as 0.2–3 cases per 100,000 individuals per year [1, 6, 7], however, in the recent years the incidence has increased significantly. Many countries now report increases of up to 7.4 per 100,000 individuals per year [5, 8–10].

Despite improved diagnostics and therapeutics, successful disease treatment remains difficult, as spondylodiscitis continues to be associated with high mortality. Not much is known about the influence of most of the detected pathogens on the clinical course of the disease. Similarly, it is not known, if pathogen detection via intraoperative tissue or blood culture makes a difference for the clinical course of the disease. A more detailed understanding of clinical courses and their specific features related to defined bacterial pathogens may help to anticipate problems and dangers at earlier time points, allowing for timelier optimization of antibiotic, and surgical therapy strategies. For this reason, we retrospectively examined a cohort of patients and analyzed the influence of microbiological diagnoses on the clinical course of spondylodiscitis.

## Methods

### Study setting/inclusion and exclusion criteria

We performed a retrospective analysis of all spondylodiscitis cases from 2014 to 2018 in our spine department, in a 1700-bed, tertiary care medical center. All community-acquired hematogenous and per continuitatem cases were included. Nosocomial and post-traumatic spondylodiscitis cases were excluded. In total, 211 patients were included into the analysis.

Electronic patient records were anonymized and evaluated for laboratory findings and clinical parameters, comorbidities, revision and mortality rate, microbiological and histopathological findings, complications (renal failure, shock, pneumonia etc.), and therapeutic measures.

The impact of the detected pathogen from intraoperative tissue specimens (localized infection) and blood cultures (infection with systemic involvement) on the course of spondylodiscitis, complications, and mortality, as well as its association with pre-existing conditions was analysed. Detected pathogens were grouped (*S. aureus,* coagulase-negative staphylococci (CNS), *Enterobacterales, Enterococcus* sp., *Streptococcus* sp., *M. tuberculosis*)*.*

### Diagnosis and treatment

Spondylodiscitis was diagnosed by classical radiological changes of the intervertebral disc and the adjacent vertebrae, as well as possible epidural abscesses using MRI. To determine osseous integrity, computed tomography was also performed. Patients not eligible for MRI diagnostics, received positron emission tomography-CT. Radiological imaging was evaluated by a radiologist and two orthopedic surgeons. According to the Infectious Diseases Society of America guidelines[11], diagnosis of spondylodiscitis was also based on the clinical parameters, such as back or neck pain, fever, and leucocyte count, inflammatory biomarker analysis [C-reactive protein (CrP), procalcitonin], pathogen detection in blood cultures and intraoperative tissue specimens, and histopathological work-up.

Microbiological diagnostic work-up included two sets of blood cultures (BD Bactec Plus aerobic and anaerobic medium) if possible drawn before start of antibiotic therapy, and analysis of 3–5 tissue specimens, collected either during open surgery or by transpedicular biopsy with a 10-gauge Jamshidi needle. Tissue specimens were homogenized upon arrival in the microbiological laboratory, and plated onto Columbia blood agar, Chocolate agar, and Sabouraud agar (all Thermo Fisher, Bremen, Germany). In addition, 2 ml Thioglycolate broth (Thermo Fisher, Bremen, Germany) was inoculated. Plates were incubated over 14 days at 37 °C in atmosphere containing 5% CO_2_. Bacteria were identified by whole-cell MALDI-TOF mass spectrometry on a Biotyper instrument (Bruker Daltonics, Bremen, Germany). Bacteria with high to medium pathogenic potential, unlikely to occur as contaminants in intraoperative specimens, such as *S. aureus*, *Enterobacterales or Enterococcus *sp. were considered significant if recovered from at least one intraoperative specimen. Low-pathogenic bacteria (e.g., CNS, *Cutibacterium* sp.) were considered clinically significant only when recovered from at least two independent intraoperative specimens.

For histopathological work-up, specimens were fixed in 10% formalin and gram and haematoxylin and eosin staining were performed. At least 15 high power fields (HPF; × 400) in five different areas of the specimen were examined and scanned for the presence of neutrophil polymorph leukocytes (NP). Mean numbers of NPs per HPF were calculated, and spondylodiscitis was considered positive if more than one NP on average per HPF was present. In suspected mycobacterial spondylodiscitis cases, Ziehl–Nielsen staining was performed and scanned for granuloma.

If histopathological and microbiological examinations were negative, but MR imaging showed clear signs compatible with spondylodiscitis and clinical and laboratory signs were present for spondylodiscitis, this was rated as spondylodiscitis. If histopathological and microbiological examinations were negative and spondylodiscitis was described as questionable on MRI, the cases were not classified as spondylodiscitis and excluded from the study.

The decision on surgical versus conservative therapy was made taking into account possible instability, the extent of osseous destruction, the presence of abscesses and neurological deficits and concomitant diseases. Conservatively treated patients received a lumbar or thoracolumbar brace for 12 weeks and antibiotic therapy. The majority of patients were treated surgically, with different surgical procedures depending on the infection location, the clinical condition of the patient and the extent of bony defects.

**Fig. 1:**
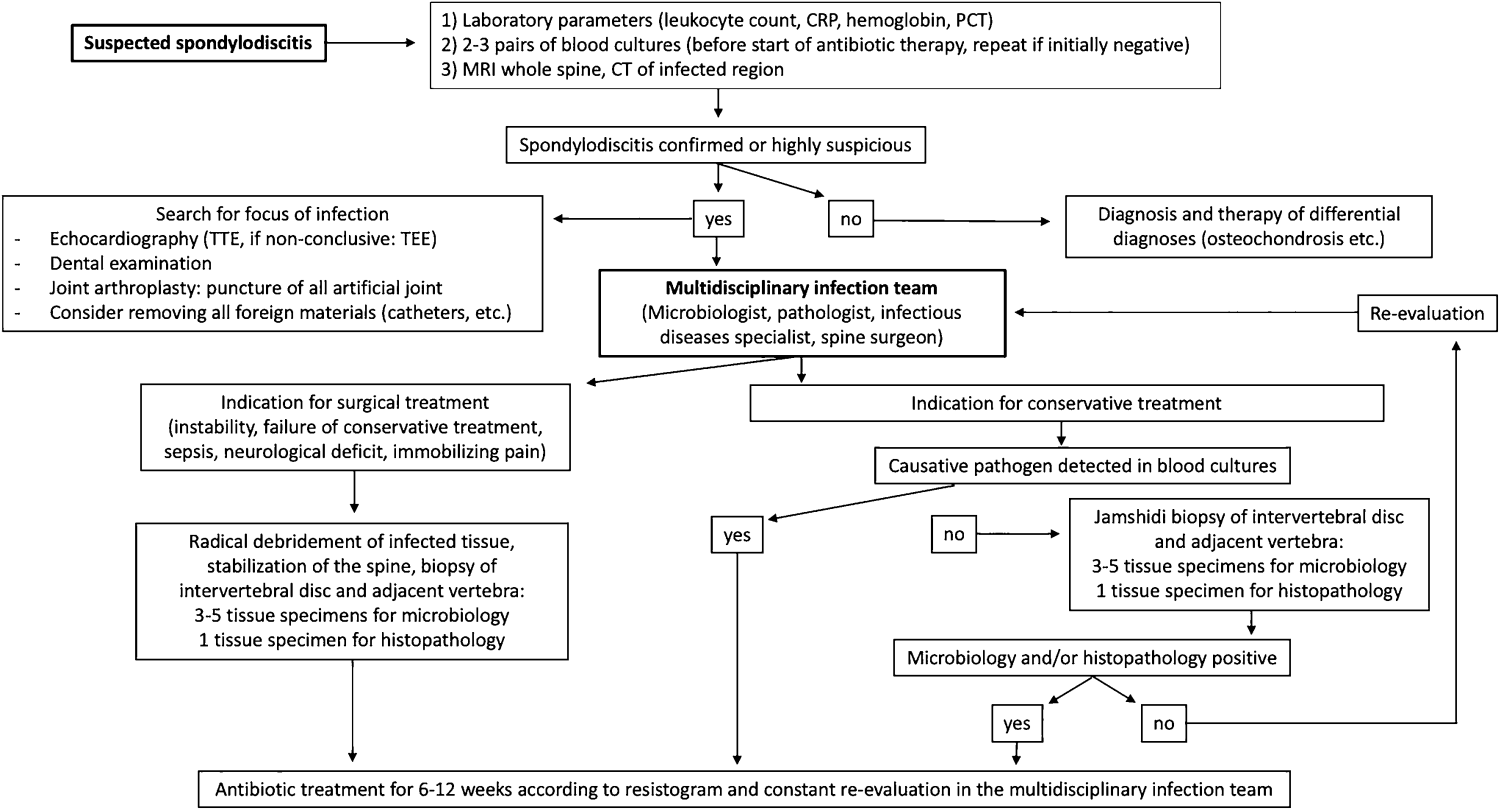
Structured approach for diagnosis and therapy of spondylodiscitis

The type and duration of antibiotic therapy were determined individually, in the context of a multidisciplinary infection board. The structured approach for diagnosis and therapy of spondylodiscitis can be found in Fig. 1.

### Statistical analysis

Statistical analyses were performed using IBM SPSS v23.0 and Graphpad Prism v8. The results were statistically significant at *p* < 0.05. Student’s *t* tests, Chi-square test, and Fisher’s exact tests were used for data comparisons. The study was conducted according to the ethics committee of the local medical chamber and the Declaration of Helsinki.

## Results

### Baseline characteristics and microbiological diagnosis

The mean patient age was 64.6 ± 14.8 years, with a range of 19–89 years. 72 patients were females (34.1%) and the average body mass index (BMI) of the cohort was 26.9 ± 15.1 kg/m^2^. The median follow-up was 144 days. 24 patients were treated conservatively with antibiotic therapy alone, of whom 15 had a percutaneous biopsy, whereas the remaining 187 were treated surgically.

In 69.2% (*n* = 146) one spinal segment was affected by the infection, 65 cases (30.8%) showed a multi-level spondylodiscitis.

In all treated patients, specimens for microbiological examination were obtained (blood cultures, *n* = 182; tissue specimens, *n* = 202). In 173 patients, blood cultures and tissue specimens were available for analysis. A histopathological sample was obtained from 172 of 202 surgically treated patients, which was positive for spondylodiscitis in 68.0% (117/172).

In 97/211 patients (46.0%), pathogenic bacteria were detected in blood culture, since in 29 patients no blood cultures were taken, detection rate was 53.3% (97/182). Likewise, a causative pathogen was detected in intraoperative samples from 134/202 cases (66.3%). In 78 cases, only intraoperative specimens were positive, in 28 cases bacteria were recovered from blood cultures, but not from intraoperative specimens. The species of blood and tissue isolates in patients where blood culture and intraoperative tissue showed positive results were concordant in 54/64 cases (84.4%; Table 1).

**Table 1:** Microbiological diagnosis overlap

Intraoperative tissue													
	MSSA	MRSA	*S. hominis*	*S. epidermidis*	*E. faecalis*	*E. faecium*	*E. coli*	*S. sanguinis*	*M. tuberculosis*	*Candida* spp.	*Klebsiella* spp.	other	Total
Blood culture													
MSSA													
*N*	29	0	1	1	0	1	0	0	0	2	1	1	36
%	87.9%	0.0%	100.0%	7.7%	0.0%	100.0%	0.0%	0.0%	0.0%	100.0%	50.0%	11.1%	
MRSA													
*N*	0	4	0	0	0	0	0	0	0	0	1	0	5
%	0.0%	100.0%	0.0%	0	0.0%	0.0%	0.0%	0.0%	0.0%	0.0%	50.0%	0.0%	
*S. epidermidis*													
*N*	0	0	0	8	0	0	0	1	0	0	0	2	11
%	0.0%	0.0%	0.0%	61.5%	0	0.0%	0.0%	50.0%	0.0%	0.0%	0.0%	22.2%	
*E. faecalis*													
*N*	0	0	0	1	3	0	0	0	0	0	0	0	4
%	0.0%	0.0%	0.0%	7.7%	75.0%	0.0%	0.0%	0.0%	0.0%	0.0%	0.0%	0.0%	
*E. faecium*													
*N*	0	0	0	0	0	0	0	0	0	0	0	1	1
%	0.0%	0.0%	0.0%	0.0%	0.0%	0.0%	0.0%	0.0%	0.0%	0.0%	0.0%	11.1%	
*E. coli*													
*N*	0	0	0	0	0	0	4	0	0	0	0	0	4
%	0.0%	0.0%	0.0%	0.0%	0.0%	0.0%	100.0%	0.0%	0.0%	0.0%	0.0%	0.0%	
*P. mirabilis*													
*N*	1	0	0	0	0	0	0	0	0	0	0	0	1
%	3.0%	0.0%	0.0%	0.0%	0.0%	0.0%	0.0%	0.0%	0.0%	0.0%	0.0%	0.0%	
*M. tuberculosis*													
*N*	0	0	0	0	0	0	0	0	1	0	0	0	1
%	0.0%	0.0%	0.0%	0.0%	0.0%	0.0%	0.0%	0.0%	100.0%	0.0%	0.0%	0.0%	
*Candida *spp.													
*N*	1	0	0	1	0	0	0	0	0	0	0	0	2
%	3.0%	0.0%	0.0%	7.7%	0.0%	0.0%	0.0%	0	0.0%	0.0%	0.0%	0.0%	
Other													
*N*	2	0	0	2	1	0	0	1	0	0	0	5	11
%	6.1%	0.0%	0.0%	15.4%	25.0%	0	0.0%	50.0%	0.0%	0.0%	0.0%	55.6%	
Total													
*N*	33	4	1	13	4	1	4	2	1	2	2	9	76

Percentages shown are fractions of intraoperative tissue results

*MSSA* methicillin-susceptible *S. aureus*, *MRSA* methicillin-resistant *S. aureus*

*S. aureus* was by far the most frequent pathogen detected in blood culture and intraoperative tissue (31.0% and 36.7%, respectively), followed by *S. epidermidis* (intraoperative tissue, 17.3%; blood culture, 23%) (Table 2). In 12 specimens and 12 blood cultures, more than one pathogen was isolated.

**Table 2:** Microbiological diagnosis

Intraoperative tissue		
*S. aureus* (methicillin-susceptible)	55	36.7%
*S. aureus* (methicillin-resistant)	4	2.7%
*S. hominis*	1	0.7%
*S. epidermidis*	26	17.3%
*E. faecalis*	4	2.7%
*E. faecium*	3	2.0%
*E. coli*	7	4.7%
*P. aeruginosa*	2	1.3%
*P. mirabilis*	1	0.7%
*S. sanguinis*	2	1.3%
*S. agalactiae*	1	0.7%
*S. mitis*	2	1.3%
*M. tuberculosis*	8	5.3%
*Candida *spp.	5	3.3%
*Klebsiella *spp.	3	2.0%
Other	26	17.3%
Total	*150*	*100.0%*
Blood culture		
*S. aureus* (methicillin-susceptible)	35	31.0%
*S. aureus* (methicillin-resistant)	6	5.3%
*S. hominis*	1	0.9%
*S. epidermidis*	26	23.0%
*E. faecalis*	7	6.2%
*E. faecium*	4	3.5%
*E. coli*	8	7.1%
*P. mirabilis*	3	2.7%
*S. agalactiae*	2	1.8%
*S. mitis*	1	0.9%
*M. tuberculosis*	1	0.9%
*Candida* spp.	4	3.5%
Other	15	13.3%
Total	113	100.0%

Of the 41 patients in whom no causative pathogen was recovered, 31 had been administered antibiotics (75.6%) prior to blood culture or intraoperative sampling. In contrast, in 170 patients with microbiological diagnosis, only 47.1% (80/170) had previously received an antibiotic (*p* = 0.001, Chi-square test).

### Key metrics by microorganism

Recovered bacterial species from blood cultures and intraoperative tissues did not affect length of hospital stay, length of ICU stay, white blood cell counts, and creatinine.

Patients with evidence of *S. aureus* in blood culture showed significantly higher preoperative CrP levels when compared with other pathogens [196 mg/l, standard deviation (SD) 121 mg/l], when compared with CNS (91 mg/l, SD = 69 mg/l, *p* = 0.003), and other bacteria (107 mg/l, SD = 86 mg/l, *p* = 0.03).

Gender had no influence on the pathogens detected. The occurrence of epidural abscesses (*n* = 98, 46.4%) and psoas abscesses (*n* = 47, 22.3%) showed no dependence on bacterial species.

Intravenous antibiotics were given on average for 27 ± 22 days, with a total duration antibiotic therapy of 69 ± 35 days. For bacterial detection in intraoperative tissue, there was a significant difference in antibiotic administration duration when *M. tuberculosis* was detected (178 days, SD 3.7, *p* < 0.001) with respect to all other bacterial groups.

### Comorbidities and complications by pathogen

**Fig. 2:**
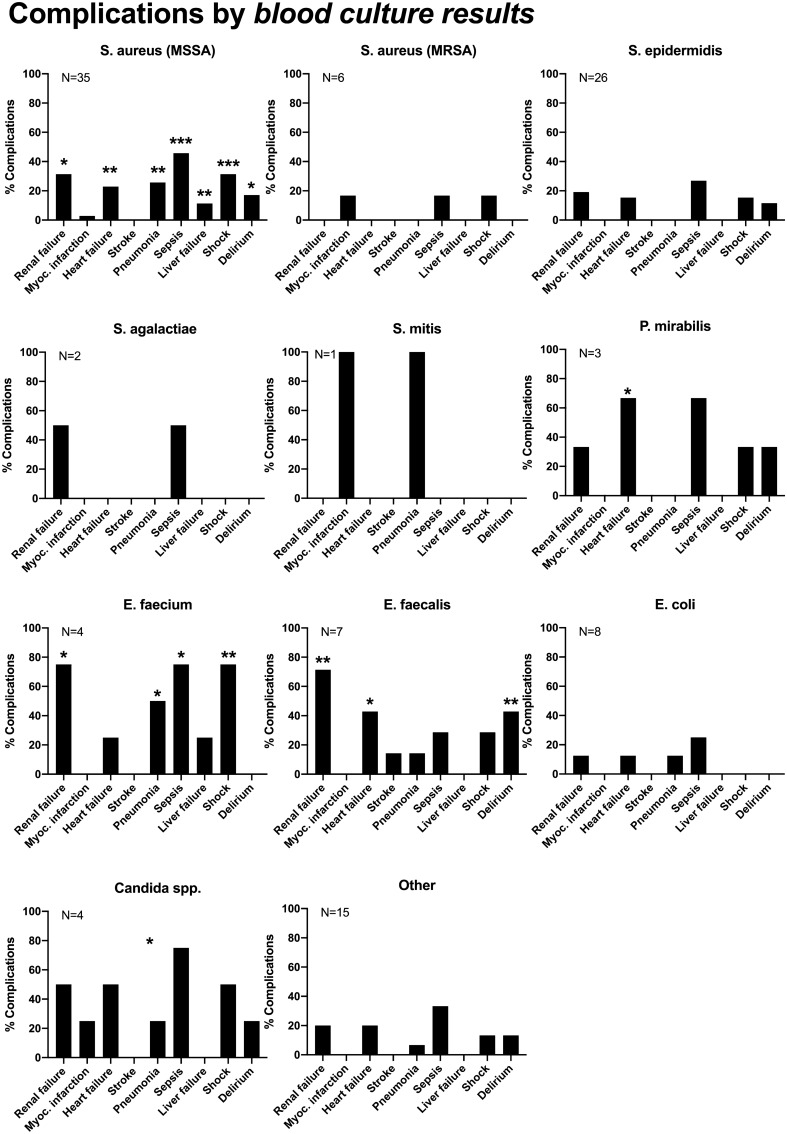
Complications by blood culture results. *p < 0.05; **p < 0.01; ***p < 0.001

**Fig. 3:**
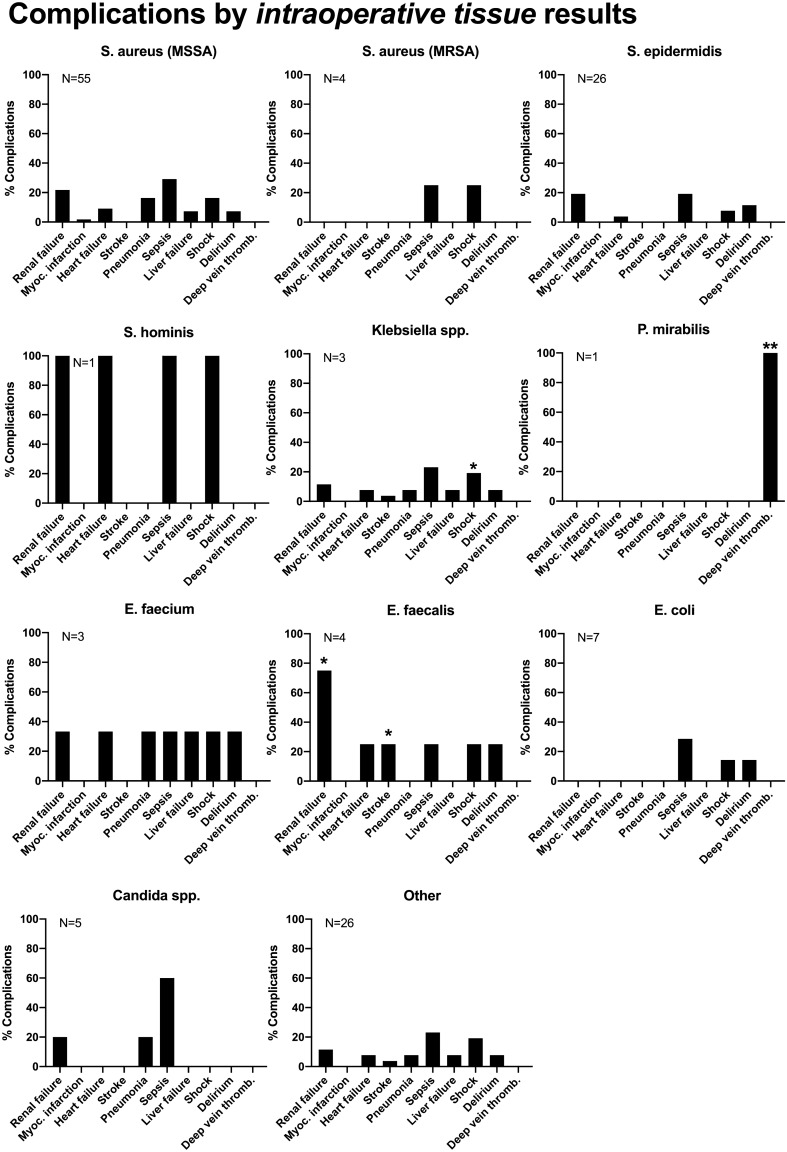
Complications by intraoperative tissue results. *p < 0.05; **p < 0.01; ***p < 0.001

The influence of the detected pathogen on inpatient complications, such as sepsis, renal failure, pneumonia, etc. for blood culture and intraoperative tissue results are listed in Figs. 2 and 3. Pre-existing comorbidities, such as malignoma, diabetes etc. were not associated with detection of specific pathogens.

### Localization of spondylodiscitis by pathogen

*S. aureus* showed a remarkable distribution pattern with regard to the localization of spondylodiscitis. The percentage of *S. aureus* detected compared to all detected bacterial species in cervical spondylodiscitis cases was significantly increased compared to other locations of the spine. In cases, where *S. aureus* was found in blood culture, locations were: cervical 31.4%, 11/35; thoracic 14.3%, 5/35; lumbar 42.9%, 15/35; multifocal 11.4%, 4/35.

Likewise, when *S. aureus* was found in intraoperative tissue, locations were: cervical 32.7%, 18/55; thoracic 14.5%, 8/55; lumbar 40.0%, 22/55; multifocal 12.7%, 7/55. Thus, spondylodiscitis caused by *S. aureus* was roughly twice as often localized in the cervical spine compared to spondylodiscitis of any other cause (blood culture: *p* = 0.026, intraoperative tissue: *p* < 0.001).

*M. tuberculosis* was significantly more frequent in thoracic regions (thoracic 75%, 6/8; lumbar 25%, 2/8; *p* = 0.004). Other bacterial species showed no significant differences in distribution, with *S. epidermidis* showing a tendency in intraoperative biopsies for disseminated spondylodiscitis cases (*p* = 0.07) (Table 3).

**Table 3:** Localization by bacteria species

	Cervical		Thoracic		Lumbar		Disseminated		*p* value
Intraoperative tissue									
*S. aureus* (methicillin-susceptible)	32.7%	18	14.5%	8	40.0%	22	12.7%	7	**< 0.001**
*S. aureus* (methicillin-resistant)	0.0%	0	75.0%	3	25.0%	1	0.0%	0	0.090
*S. hominis*	0.0%	0	0.0%	0	100.0%	1	0.0%	0	0.828
*S. epidermidis*	15.4%	4	7.7%	2	57.7%	15	19.2%	5	0.070
*E. faecalis*	0.0%	0	25.0%	1	50.0%	2	25.0%	1	0.583
*E. faecium*	33.3%	1	33.3%	1	33.3%	1	0.0%	0	0.743
*E. coli*	14.3%	1	0.0%	0	85.7%	6	0.0%	0	0.293
*P. aeruginosa*	0.0%	0	50.0%	1	50.0%	1	0.0%	0	0.765
*P. mirabilis*	0.0%	0	0.0%	0	100.0%	1	0.0%	0	0.828
*S. sanguinis*	0.0%	0	0.0%	0	100.0%	2	0.0%	0	0.618
*S. agalacatiae*	0.0%	0	0.0%	0	100.0%	1	0.0%	0	0.828
*S. mitis*	50.0%	1	0.0%	0	50.0%	1	0.0%	0	0.540
*M. tuberculosis*	0.0%	0	75.0%	6	25.0%	2	0.0%	0	**0.004**
*Candida *spp.	40.0%	2	40.0%	2	20.0%	1	0.0%	0	0.250
*Klebsiella *spp.	0.0%	0	33.3%	1	66.7%	2	0.0%	0	0.803
Other	11.5%	3	15.4%	4	65.4%	17	7.7%	2	0.594

Blood culture									
*S. aureus* (methicillin-susceptible)	31.4%	11	14.3%	5	42.9%	15	11.4%	4	**0.026**
* S. aureus* (methicillin-resistant)	16.7%	1	50.0%	3	33.3%	2	0.0%	0	0.392
* S. hominis*	0.0%	0	100.0%	1	0.0%	0	0.0%	0	0.332
* S. epidermidis*	3.8%	1	15.4%	4	69.2%	18	11.5%	3	0.164
* E. faecalis*	14.3%	1	14.3%	1	42.9%	3	28.6%	2	0.285
* E. faecium*	25.0%	1	50.0%	2	25.0%	1	0.0%	0	0.473
* E. coli*	0.0%	0	25.0%	2	62.5%	5	12.5%	1	0.657
* P. mirabilis*	0.0%	0	33.3%	1	66.7%	2	0.0%	0	0.803
* S. agalactiae*	0.0%	0	0.0%	0	100.0%	2	0.0%	0	0.618
* S. mitis*	0.0%	0	0.0%	0	100.0%	1	0.0%	0	0.828
* M. tuberculosis*	0.0%	0	100.0%	1	0.0%	0	0.0%	0	0.332
* Candida *spp.	0.0%	0	25.0%	1	75.0%	3	0.0%	0	0.714
Other	13.3%	2	20.0%	3	60.0%	9	6.7%	1	0.956
Overall distribution of spondylodiscitis	15.6%	33	22.7%	48	53.1%	112	8.5%	18	

Statistical testing using Chi-squared test

### Patient mortality by pathogen

Patients in which *S. aureus* was isolated from blood cultures showed a significantly higher mortality of 31.4% (11/35, *p* = 0.001) as compared to 13.7% in the overall cohort (Table 4).

**Table 4:** Mortality by bacteria species

	*N*	Death	%	*p* value
Intraoperative tissue				
*S. aureus* (methicillin-susceptible)	55	10	18.2%	0.182
*S. aureus *(methicillin-resistant)	4	1	25%	0.447
*S. hominis*	1	1	100%	0.137
*S. epidermidis*	26	4	15.4%	0.491
*E. faecalis*	4	0	0%	0.553
*E. faecium*	3	2	66.7%	**0.050**
*E. coli*	6	1	14.3%	0.649
*P. aeruginosa*	2	1	50%	0.255
*P. mirabilis*	1	0	0%	0.863
*S. sanguinis*	2	0	0%	0.745
*S. agalactiae*	1	0	0%	0.863
*S. mitis*	2	0	0%	0.745
*M. tuberculosis*	8	0	0%	0.302
*Candida *spp.	5	1	20%	0.524
*Klebsiella *spp.	3	2	66.7%	**0.050**
Other	26	6	23.1%	0.121
*Blood culture*				
*S. aureus* (methicillin-susceptible)	35	11	31.4%	**0.002**
*S. aureus *(methicillin-resistant)	6	1	16.7%	0.591
*S. hominis*	1	0	0%	0.863
*S. epidermidis*	26	3	11.5%	0.509
*E. faecalis*	7	0	0%	0.351
*E. faecium*	4	2	50.0%	0.091
*E. coli*	8	1	12.5%	0.699
*P. mirabilis*	3	3	100%	**0.002**
*S. agalactiae*	2	0	0%	0.745
*S. mitis*	1	0	0%	0.863
*M. tuberculosis*	1	0	0%	0.863
*Candida *spp.	4	1	25%	0.447
Other	15	5	33.3%	**0.038**

Statistical testing using Fisher’s Exact test

## Conclusions

Comprehensive microbiological analysis plays a crucial role in the treatment of spondylodiscitis. Effective antibiotic treatment relies on the detection of the causative organisms and its susceptibility testing. To achieve this, at least two peripheral blood cultures as well as multiple biopsies should be sent for microbiological analysis [11].

Bacterial detection in spondylodiscitis is successful in approximately 50–83% of cases [3, 12]. In this study, we detected bacteria in intraoperative samples or blood cultures in 170/211 (80.6%) patients. In combination with histopathology, we increased the rate of definitive diagnosis in suspected spondylodiscitis cases to 86.7% (*n* = 183/211).

In 28 of 211 patients (13.3%), the determination of the pathogen was only possible through blood cultures, which underlines the great importance of the blood cultures for the diagnosis of spondylodiscitis and its targeted antibiotic therapy.

MSSA was the most commonly detected pathogen, in both intraoperative (31%) and blood cultures (36.7%), followed by *S. epidermidis*, as has been found in previous studies (28–55%) [13–17].

Concordance of blood cultures and intraoperative samples was 84.3% (54/64), whereas Bae et al. reported a significantly higher concordance (95.7%, 135/141) [18]. Our lower concordance could be due to the fact that our analysis included positive blood cultures that were applied during the entire in-patient stay, while Bae et al. only included blood culture from before start of therapy. These included those that were found during an intensive care unit stay and could possibly be caused by bacteremia of another origin (pulmonary, urinary tract etc.). Importantly, *CNS* from intraoperative samples showed a much lower concordance of 57.1% (8/14) with results from blood culture indicating possible contamination, thereby adversely affecting overall concordance.

Our results demonstrated that the start of antibiotic therapy influenced the detection rate of pathogens. I* n* > 75% of patients in whom no causative pathogen was detected, antibiotic therapy was started prior to sampling, while this was the case in less than 50% of patients with definitive microbiological diagnosis. This observation supports the suggestion that blood cultures and intraoperative specimens should be obtained prior to antibiotic administration [19].

In our patients, a clear preference by MSSA for the cervical spine over other localizations was shown (32.2% vs. 15.6% overall). MSSA was the causative pathogen in 62% of cervical spondylodiscitis cases. Other authors found MSSA in 56 and 72% of cervical spondylodiscitis, supporting our observation [20, 21]. The reasons why MSSA frequencies are higher in the cervical spine remain unclear and require further investigation. The identification of *M. tuberculosis* in the thoracic spine of 75% of cases is in agreement with distribution patterns of tuberculous spondylitis in the literature [22].

In our collective, epidural abscesses were found in 26 of 33 cervical spondylodiscitis cases (78.8%) compared with 46.4% (98/211) for the overall cohort (*p* < 0.001).

Other studies have demonstrated increased general risks of developing epidural abscesses in MSSA or Gram-positive bacteria against gram-negative bacteria with *S. aureus* expressing virulence factors that promote abscess formation [23–25]. In our collective, only for the cervical spine, however, a connection between MSSA and the occurrence of epidural abscesses could be demonstrated, since 16 out of 18 (88.9%) cases of cervical spondylodiscitis caused by MSSA (detection from intraoperative samples) showed epidural abscesses.

MSSA impacted on in-patient complications when recovered from blood culture, supporting the work by Loibl et al*.* in their small patient population [17]. We found a significantly increased incidence of patient acute renal failure, cardiac decompensation, pneumonia, sepsis, liver failure, shock, and postoperative delirium for MSSA detection, when compared with other bacteria. Inpatient and intensive care unit stay, however, showed no dependency. There are indications of increased complications and mortality in spondylodiscitis caused by *Enterococcus sp*. and *Enterobacterales,* but due to very small numbers no definite interpretation is possible. The data on these bacteria and spondylodiscitis are entirely lacking in the literature. However, the length of stay in the hospital and in the intensive care unit showed no dependence on the microbiological diagnosis.

Although intraoperative detection of MSSA showed an increased mortality of 18.2% (10/55), when compared to overall mortality of 13.7% (29/211), this was not statistically significant (*p* = 0.259). Also, when MSSA was detected in blood cultures, mortality increased by a factor of 2.3, and thus became highly significant (*p* = 0.001). This observation was consistent with increased complication rates in systemic MSSA infection, and was in agreement with other studies demonstrating increased mortality from MSSA bacteremia, and studies which identified MSSA bacteremia as a risk factor for treatment failures from antibiotic therapy [17, 26, 27]. In contrast, Kehrer and Park did not find increased mortality in MSSA spondylodiscitis, although no distinction was made between intraoperative detection and positive blood culturing [4, 25].

The frequent occurrence of complications and, in particular, the increased mortality from bacteremia with *S. aureus* in our study again emphasize the importance of blood cultures in the diagnostic process.

This study has some limitations, e.g., the retrospective design in a single-center, and the lack of a longer follow-up period with clinical scores. Although this study has a relatively high number of patients compared with the literature, the number of cases caused by rarer pathogens, apart from *S. aureus* and *S. epidermidis,* were relatively small, therefore, the informative value with regard to these species is limited. Another limitation is that the positive detection of blood cultures in rare cases could also be caused by other foci of infection (e.g., infected central venous catheters, pneumonia, etc.) of this very sick patient population that existed at the same time.

In conclusion, this study provides evidence that *S. aureus* preferentially infects the cervical spine, and that spondylodiscitis caused by *S. aureus* results in more severe clinical symptoms, with an increased mortality rate. In future studies, a prospective study design and larger case numbers will be necessary to ascertain differences between specific pathogens, which could have an impact on composition and duration of antibiotic therapies and surgical strategies.
